# Infective Endocarditis: A Contemporary Review of Epidemiology, Diagnosis, and Management

**DOI:** 10.3390/antibiotics15050482

**Published:** 2026-05-09

**Authors:** Angela Ishak, Yusuf Kamran Qadeer, Mousa Mahmoud AlRawashdeh, Bing Yue, Muzamil Khawaja, Markus Strauss, Chayakrit Krittanawong

**Affiliations:** 1 Department of Internal Medicine, Henry Ford Hospital, Detroit, MI 48202, USA; aishak1@hfhs.org (A.I.); malraw1@hfhs.org (M.M.A.); 2 Division of Cardiology, Henry Ford Hospital, Detroit, MI 48202, USA; yqadeer@umich.edu; 3 Peaceheart Southwest Medical Center, Vancouver, WA 98664, USA; byue@peacehealth.org; 4 Department of Cardiology, Emory University, Atlanta, GA 30322, USA; ahmed220@gmail.com; 5 Department of Cardiology I-Coronary and Peripheral Vascular Disease, Heart Failure Medicine, University Hospital Muenster, Cardiol, 48149 Muenster, Germany; 6 HumanX, Delaware, DE 19958, USA

**Keywords:** endocarditis, infective endocarditis, prosthetic valve endocarditis, antimicrobial therapy, valve surgery

## Abstract

Infective endocarditis (IE) is an uncommon but life-threatening condition characterized by infection and inflammation of the endocardial surface of the heart, most commonly affecting native or prosthetic valves. Recent data indicate in-hospital mortality rates ranging from 15% to 25%, with evidence of increasing mortality even in high-income countries. Beyond its fatal potential, IE poses a major public health burden, accounting for over 1.7 million disability-adjusted life years (DALYs) globally in 2019. This review aims to discuss recent advancements in the diagnosis and management of IE given the shifting epidemiology and pathogen profile of the disease. There is a rising incidence of healthcare-associated IE and an expanding population of vulnerable patients, including the elderly and those with prosthetic material or indwelling catheters. Diagnostic capabilities have rapidly advanced with the adoption of modalities such as 18F-fluorodeoxyglucose positron emission tomography/computed tomography (FDG-PET/CT), cardiac CT, and cardiac magnetic resonance (CMR), particularly in patients with prosthetic valve endocarditis or culture-negative presentations. Additionally, the expanding indications for surgical intervention and increasing antimicrobial resistance have added complexity to management decisions. These developments underscore the need for a comprehensive review to support healthcare providers in navigating the modern diagnostic and therapeutic landscape of IE.

## 1. Introduction

Infective endocarditis (IE) is an uncommon but life-threatening condition characterized by infection and inflammation of the endocardial surface of the heart, most commonly affecting native or prosthetic valves. The global incidence of IE is estimated to range between 3 and 7 cases per 100,000 person-years, and it remains a condition associated with substantial morbidity and mortality [1,2]. Recent data indicate in-hospital mortality rates ranging from 15% to 25%, with evidence of increasing mortality even in high-income countries [1,3]. Beyond its fatal potential, IE poses a major public health burden, accounting for over 1.7 million disability-adjusted life years (DALYs) globally in 2019 [3].

While the overall incidence has remained relatively stable, the epidemiologic profile of IE has undergone significant shifts. Recent population-based studies show a rising median age of affected patients, now nearing 73 years, reflecting a growing burden in older populations [4]. The decline in rheumatic heart disease as a predisposing factor has been counterbalanced by increases in degenerative valvular disease, diabetes mellitus, congenital heart disease, and intravenous drug use [4,5]. Notably, healthcare-associated IE—often linked to prosthetic devices, intravascular catheters, and dialysis—has become increasingly prevalent and is associated with higher mortality and worse clinical outcomes compared to community-acquired infections [6]. These changes underscore the evolving complexity of IE diagnosis, treatment, and prevention in modern medical practice.

The microbiological profile of IE has evolved considerably over time. *Staphylococcus aureus* has now emerged as the most identified pathogen worldwide, responsible for approximately 26–30% of all cases, and it is notably associated with healthcare-related infections and intravenous drug use [3,7]. In comparison, the viridans group streptococci, once the predominant cause in the pre-antibiotic era, now account for around 18% of cases, largely in community-acquired settings [2,7]. Enterococcus species, especially *Enterococcus faecalis*, have also risen in significance, particularly among elderly and hospitalized patients with genitourinary or gastrointestinal comorbidities [3,7]. The HACEK organisms (*Haemophilus*, *Aggregatibacter*, *Cardiobacterium*, *Eikenella*, and *Kingella*) remain relatively rare but important, especially in culture-negative cases [7]. Importantly, healthcare-associated IE is increasingly caused by multidrug-resistant pathogens, including methicillin-resistant *Staphylococcus aureus* (MRSA) and resistant gram-negative bacilli, often leading to delayed clearance of bacteremia and worse outcomes [6,8,9].

This review aims to discuss recent advancements in the diagnosis and management of IE given the shifting epidemiology and pathogen profile of the disease. As discussed above, there is a rising incidence of healthcare-associated IE and an expanding population of vulnerable patients, including the elderly and those with prosthetic material or indwelling catheters. Diagnostic capabilities have rapidly advanced with the adoption of modalities such as 18F-fluorodeoxyglucose positron emission tomography/computed tomography (FDG-PET/CT), cardiac CT, and cardiac magnetic resonance (CMR), particularly in patients with prosthetic valve endocarditis or culture-negative presentations [2,10]. Additionally, the expanding indications for surgical intervention and increasing antimicrobial resistance have added complexity to management decisions. These developments underscore the need for a comprehensive review to support healthcare providers in navigating the modern diagnostic and therapeutic landscape of IE.

## 2. Predisposing Risk Factors for Infective Endocarditis

### 2.1. Cardiac-Specific Risk Factors

Several pre-existing cardiac conditions significantly increase the risk of IE. Patients with prosthetic heart valves, both mechanical and bioprosthetic, are at elevated risk, with prosthetic valve endocarditis accounting for over 20% of all IE cases in a large, multinational cohort of patients with IE [5]. Moreover, retrospective cohort studies from Denmark and Finland have also demonstrated that among patients undergoing prosthetic heart valve replacements, whether surgical or transcatheter, the incidence of IE ranges from approximately 3 to 10 cases per 1000 person-years [11,12]. In particular, transcatheter valve procedures, such as transcatheter aortic valve replacement (TAVR), can be complicated by IE [13]. IE following TAVR is associated with high mortality (30-day mortality rate of 18.5%, one-year mortality rate of 45.6%) and risk for stroke, especially within the first year of implantation [13,14]. The most common pathogens causing IE following TAVR are *Staphylococcus aureus* (22%), *Streptococcus* species (20%), and *Enterococcus* species (15.5%) [14]. Several factors increase the risk for TAVR-IE, including younger age at the time of TAVR, male sex, prior history of endocarditis, liver and lung disease, and post-TAVR acute kidney injury [14]. Surgical management of TAVR-IE is often complicated due to the high-risk profile of the patients and often requires a multidisciplinary approach [15].

Another major risk factor is a history of prior IE, with studies suggesting recurrence rates of approximately 5% [16]. Cardiac implantable electronic devices (CIEDs), including pacemakers and implantable cardioverter defibrillators (ICDs), are also associated with rising rates of device-related endocarditis, likely due to lead infections or bacteremia in the setting of long-term hardware placement [17].

Congenital heart disease (CHD), particularly unrepaired or partially repaired cyanotic lesions, carries a high lifetime risk of IE, ranging from 2 to 8.5% in some cohorts, with the highest risk observed in individuals with unrepaired or complex cyanotic CHD [18,19]. Bicuspid aortic valve (BAV), although present in less than 2% of the general population, is disproportionately represented in IE cases due to turbulent flow and valve degeneration, with a recent meta-analysis showing a 12-fold increase in IE in people with BAV compared to the general population [20,21]. Similarly, mitral valve prolapse (MVP), especially with associated mitral regurgitation, is a well-established risk factor, with a recent population-based study from the US estimating an incidence of IE of 87 cases per 100,000 person-years among MVP patients [22]. Finally, rheumatic heart disease, while less common in developed nations, remains a concern in vulnerable populations and immigrants from endemic areas and is an important predisposing factor for IE [23,24].

### 2.2. Systemic and Non-Cardiac Risk Factors

In addition to structural heart disease, multiple non-cardiac factors are associated with increased risk of IE. Intravenous drug use (IVDU) remains a leading cause across the developed world, with studies from North America indicating that rates of IE among persons who inject drugs are estimated to be 100 times higher than in non-users [25]. Chronic kidney disease (CKD), particularly in patients receiving hemodialysis, confers a 50- to 70-fold higher risk of IE compared to the general population due to repeated need for vascular access (arterio-venous grafts and indwelling catheters) and the use of immunosuppressants in renal transplant recipients [26]. Chronic liver disease, including cirrhosis, has also been associated with increased IE risk, possibly due to systemic inflammation and impaired immune responses [27].

Other important risk factors include advanced age, which is highlighted in a recent systematic review, showing that the elderly (over 60 years of age) have a fivefold increased risk of IE when compared to the general population [28]. Additionally, immunocompromised states such as HIV infection, people with malignancies, or chronic corticosteroid use further reduce host defenses against bloodstream infections [29,30]. Moreover, poorly controlled diabetes mellitus is common in patients with IE and is associated with both increased incidence and a poorer prognosis [8]. Lastly, indwelling central venous catheters remain a key contributor to healthcare-associated IE, particularly in critically ill patients, those with malignancy, or those on dialysis [31].

## 3. Diagnosis of Infective Endocarditis

### 3.1. Clinical Suspicion

IE presents a diagnostic challenge due to its highly variable clinical manifestations, ranging from acute to subacute or chronic presentations depending on the causative organism and the patient’s underlying comorbidities. Fever is the most common symptom, reported in up to 90% of cases, followed by chills (40–75%), malaise (50–70%), fatigue (25–80%), anorexia (25–50%), weight loss (25–35%), and night sweats (25–35%) [2,5,30,32]. A new or changing cardiac murmur is present in approximately 85% of patients [33]. Classical peripheral signs, such as splinter hemorrhages, Janeway lesions, Osler nodes, and Roth spots, are less common but remain important diagnostic clues, particularly in subacute presentations [33]. More commonly, IE presents with complications such as congestive heart failure, which can occur in approximately 28.5% of patients with left-sided IE, or ischemic strokes from septic emboli, which can occur in up to 20–40% of patients with left-sided IE [34,35].

Clinicians should always maintain a high index of suspicion for IE in patients presenting with sepsis or fever of unknown origin, especially when accompanied by risk factors such as prosthetic heart valves, intracardiac devices, a history of intravenous drug use, hemodialysis dependence, or structural heart disease [36]. Scenarios that should immediately raise concern for IE include fever with a new murmur, positive blood cultures without a clear source, embolic stroke of unknown cause, and unexplained systemic embolization such as splenic or renal infarcts, limb ischemia, or mesenteric ischemia [36]. Early recognition is critical, as diagnostic delays have been consistently linked to worse outcomes and increased mortality.

### 3.2. Diagnostic Criteria

Establishing a definitive diagnosis of IE requires a structured integration of microbiological, clinical, and imaging data. The Duke Criteria have served as the standard diagnostic tool, originally developed in 1994 and revised in 2000 to improve sensitivity and specificity, particularly for blood culture-negative and prosthetic valve cases [37,38]. These criteria classify IE as “definite,” “possible,” or “rejected” based on the presence of major and minor criteria, which include microbiologic findings, evidence of endocardial involvement, and specific clinical features [37,38].

Given the evolving pathogen profiles and advances in diagnostic modalities, the 2023 Duke–ISCVID (the International Society for Cardiovascular Infectious Diseases) Criteria have been introduced with substantial updates. These include the addition of [^18F] FDG PET/CT and cardiac CT as major criteria for prosthetic valve and cardiac device-associated IE, enhancing sensitivity in this previously challenging population. Molecular diagnostics, such as valve PCR and broad-range 16S rRNA gene sequencing, are now recognized as major criteria when they confirm organisms like *Coxiella burnetii*, *Bartonella* spp., or *Tropheryma whipplei* in resected tissue or emboli [31]. The revised criteria also expand the list of typical microorganisms to include *Staphylococcus lugdunensis*, *Enterococcus faecalis*, and previously excluded genera such as *Granulicatella*, *Abiotrophia*, and *Gemella*, with additional organisms like coagulase-negative staphylococci, *Cutibacterium acnes*, and nontuberculous mycobacteria now considered typical in the setting of intracardiac prosthetic material. Furthermore, the updated criteria remove earlier requirements regarding blood culture collection timing and site separation, instead emphasizing the diagnostic value of obtaining multiple positive cultures regardless of timing or venipuncture site to better reflect real-world clinical practice [39]. Finally, the 2023 criteria introduce intraoperative surgical findings such as vegetations, valve destruction, abscesses, and prosthetic valve dehiscence as a new major criterion when other definitive diagnostic elements are unavailable [39]. These revisions have been externally validated and shown to improve diagnostic sensitivity without significantly compromising specificity. In one multicenter analysis, the 2023 Duke–ISCVID criteria demonstrated a sensitivity of 84.2% and specificity of 93.9% compared to 74.9% and 94.9%, respectively, using the modified 2000 Duke criteria, a statistically significant improvement in diagnostic yield [40].

### 3.3. Microbiologic Evaluation

Accurate microbiologic identification is paramount in the diagnosis and management of IE. Blood cultures remain the cornerstone of microbiologic evaluation, providing critical information for both diagnosis and antimicrobial therapy. In acute presentations, at least three sets of blood cultures should be obtained over a short time interval (e.g., within 1–2 h) prior to antibiotic initiation [36,41], whereas in subacute presentations, collections may be spaced over several hours to optimize diagnostic yield, and antibiotics may be withheld pending culture results [2,42]. According to the 2023 Duke–ISCVID criteria, microorganisms that commonly cause IE isolated from ≥2 separate blood culture sets, or microorganisms that occasionally or rarely cause IE isolated from ≥3 separate blood culture sets, constitute a major microbiologic criterion [39]. In fact, microbiologic findings contribute to both major and minor Duke–ISCVID criteria [40]. Automated culture systems have improved the detection of fastidious organisms, with most pathogens growing within 5 days; however, prolonged incubation may be required for slow-growing pathogens [40,43]. The most frequently isolated organisms in IE include *Staphylococcus aureus*, viridans group streptococci, *Enterococcus faecalis*, and members of the HACEK group. Recent epidemiologic trends show that *S. aureus* is now the predominant pathogen in many regions, especially among people who inject drugs and those with healthcare-associated infections [44]. Coagulase-negative staphylococci (CoNS) are more frequently implicated in prosthetic valve endocarditis [44].

Culture-negative infective endocarditis (CNIE) refers to cases of IE in which no causative organism is identified in blood cultures, despite appropriate sampling and incubation. This may occur due to prior antibiotic exposure, infection with fastidious or intracellular organisms, or inadequate microbiologic techniques [2]. Between 2.5% and 31% of IE cases are culture-negative, often due to prior antibiotic exposure or infection with fastidious or intracellular organisms such as *Coxiella burnetii*, *Bartonella* spp., and *Tropheryma whipplei* [43,45]. In such cases, serologic testing for *C. burnetii* and *Bartonella* spp. can aid diagnosis, while valve tissue PCR and 16S rRNA gene sequencing have become indispensable tools, especially in patients with prior valve surgery or embolic complications [45]. These molecular methods significantly increase diagnostic yield in culture-negative IE. Other recent advances in microbiologic diagnostics include matrix-assisted laser desorption/ionization time-of-flight mass spectrometry (MALDI-TOF MS), which enables species identification from positive cultures [46,47]. Additionally, metagenomic next-generation sequencing (mNGS) and other culture-independent methods detect microbial nucleic acids in clinical specimens, allowing broad pathogen detection [48]. However, their clinical use is limited by cost, turnaround time, and access.

**Figure 1:**
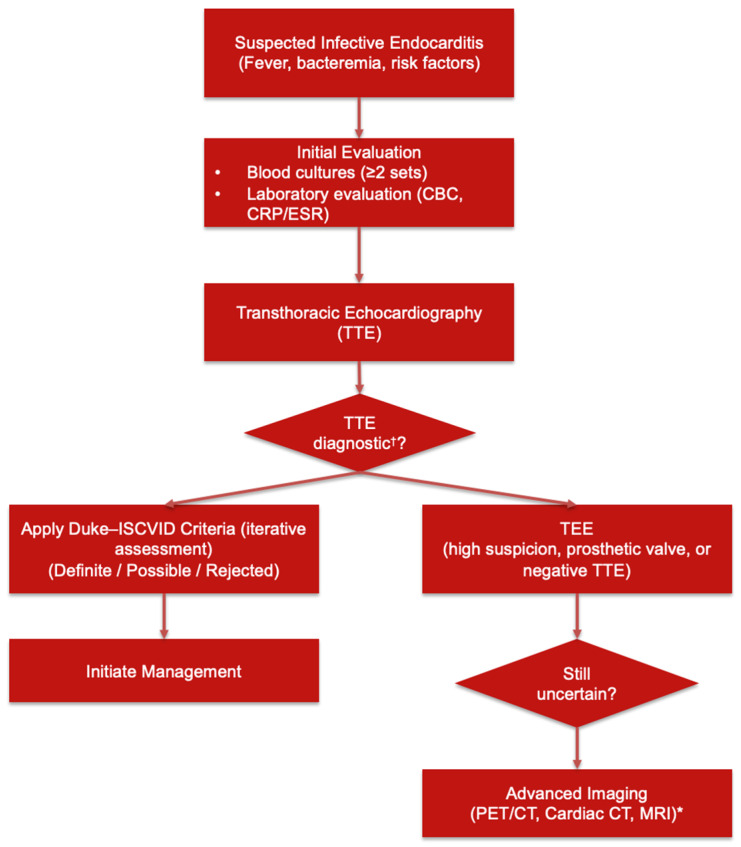
Diagnostic approach to suspected infective endocarditis. * *PET/CT* has higher diagnostic utility in prosthetic valve and device-related IE than native valve IE. ^†^ Consider transesophageal echocardiography (TEE) as the initial imaging modality in patients with prosthetic valves, cardiac implantable electronic devices (CIEDs), or suspected complicated infective endocarditis.

A simplified diagnostic algorithm integrating clinical assessment, microbiologic testing, imaging modalities, and application of the Duke–ISCVID criteria is shown in Figure 1.

## 4. The Role of Cardiac Imaging

Cardiac imaging plays a pivotal role in diagnosing and aiding in the management approach for IE and often requires a multimodal approach due to the wide variability in disease presentation.

### 4.1. Echocardiography

TTE (transthoracic echocardiogram) is typically the initial imaging modality in patients with suspected IE due to accessibility and its non-invasive nature. The use of TTE is crucial when IE is first suspected, as it helps in the identification of = vegetations and the presence of any abscesses [49]. Furthermore, it allows the assessment of the location, shape, size, and mobility of vegetations, which is key to predicting the risk of embolization in patients and therefore guiding clinical decision-making [49]. TTE is instrumental in monitoring treatment response to antibiotics in IE patients by assessing any changes in vegetations and monitoring any new complications that could indicate treatment failure and the need for surgical intervention [33].

Three-dimensional transesophageal echocardiography (3D TEE) further enhances visualization of valvular structures, allowing improved morphological characterization of vegetations and more accurate assessment of complications compared with conventional two-dimensional imaging [50,51]. In prosthetic valve endocarditis (PVE), 3D TEE is particularly useful for detecting valve perforation, dehiscence, and intracardiac fistulae [52,53], and it may influence surgical decision-making by identifying complications missed on 2D imaging. However, its lower temporal and lateral resolution compared with 2D echocardiography may result in overestimation of vegetation size [36].

The use of TEE is recommended when the findings of TTE are equivocal but clinical suspicion for IE remains significant in the case of suspected complications and in the presence of intracardiac devices [49,54]. TEE has a higher sensitivity in detecting vegetations compared to TTE, and the difference is more notable when dealing with smaller vegetations, patients with suspicion for prosthetic valve endocarditis (50% vs. 92%) or native valve endocarditis (70% vs. 96%), and patients with CIEDs [54]. Moreover, TEE is superior to TTE in detecting various IE complications, such as abscesses, where the former has shown 90% sensitivity compared to 50% in TTE [33].

### 4.2. Cardiac Computed Tomography (CT)

Cardiac CT has been proven to be especially useful in identifying perivalvular complications in IE patients when echocardiographic findings were equivocal [55]. A study revealed that cardiac CT demonstrated a sensitivity of 81% in detecting complications such as abscesses, fistulas, and pseudoaneurysms compared to 63% sensitivity with echocardiography [55]. Cardiac CT is also particularly useful when dealing with cases of prosthetic material causing shadowing in TEE, yielding inconclusive results [56]. Furthermore, cardiac CT has been utilized as part of preoperative planning, as it provides visualization of valvular anatomy and can identify abscess extension into surrounding structures, aiding in surgical decision-making [57]. Finally, cardiac CT provides superior spatial resolution compared with echocardiography for the detection of perivalvular complications, including abscesses, pseudoaneurysms, and fistulae, particularly in PVE, where echocardiographic imaging may be limited by acoustic shadowing [53,55,58]. However, cardiac CT has lower temporal resolution and is less sensitive than TEE for detecting small vegetations and leaflet perforations [58,59]. Unlike echocardiography, CT is less affected by operator variability and prosthetic material artifacts, though it is limited by radiation exposure, contrast nephrotoxicity, and availability [36,42]. Reflecting its growing diagnostic role, the 2023 Duke–ISCVID criteria now recognize characteristic findings on cardiac CT, including vegetations, abscesses, pseudoaneurysms, and intracardiac fistulae, as a major imaging criterion [39].

### 4.3. Cardiac MRI (CMR)

Although CMR is not a primary imaging modality in cases of IE, it can play a valuable role when echocardiographic findings are nonconclusive [60]. CMR may be used to identify and characterize valvular vegetations, and it is also useful in detecting complications of IE, such as abscesses, pseudoaneurysms, and embolic lesions [61]. In the absence of vegetations, delayed enhancement on CMR following contrast injection suggests endothelial damage or inflammation and can contribute to the diagnosis and management of IE [62]. Similarly, delayed enhancement can provide insight into the extension of inflammation to other nearby structures, such as the aortic wall, paravalvular tissue, cardiac chambers, and pulmonary artery [62]. Despite its usefulness in a variety of cases, its utility in IE can be limited due to susceptibility to artifacts from prosthetic materials, impacting image quality [63].

### 4.4. Cardiac PET (Positron Emission Tomography) Scan

PET scans allow identification of infections by detecting increased metabolic activity at sites of infection within the heart [64]. 18F-FDG PET/CT is recommended as part of the diagnostic evaluation of IE in patients with suspected prosthetic valve infection when echocardiographic findings are inconclusive or equivocal (Class I recommendation, ESC 2023) [65,66]. It also plays an important role in the evaluation of CIED infections. A particularly key feature of PET is its ability to offer a whole-body scan rather than just the heart, useful to investigate supportive findings of IE [65]. Although increased FDG uptake can be nonspecific due to various metabolic factors that may contribute to increased activity, signs such as increased FDG uptake in the spleen and bone marrow can be supportive of diagnosing IE [65]. Moreover, as septic dissemination of the infection can occur in various parts of the body, localizing the primary source may be challenging [67,68]. Therefore, FDG-PET/CT can provide primary source identification or portal of entry, aiding in targeted management and preventing IE recurrence [68]. A 2023 meta-analysis has revealed that 18F-FDG PET/CT has a sensitivity of 85.5% and specificity of 86% in diagnosing PVE [69]. However, due to NVEs being typically smaller in size compared to PVEs, their detection by 18F-FDG-PET may be hindered [70].

## 5. Complications Associated with Infective Endocarditis

One of the most frequent and life-threatening complications of IE is embolic events, occurring in approximately 20–30% of patients, with the highest risk observed early in the course of infection and decreasing after initiation of effective antimicrobial therapy [71,72,73]. In patients with vegetation exceeding 10mm, the risk for embolic events is significantly increased [74]. Additional predictors of embolization include vegetation mobility, prior embolic events, involvement of the mitral valve, and infection with *Staphylococcus aureus* [72,73]. The brain and spleen are the most common sites for embolic events, more notably in left-sided endocarditis [75,76]. Neurological complications, most commonly due to ischemic stroke, could occur in nearly 21.2% of IE patients, with brain abscess occurring in 1–7% of patients [75]. Splenic embolization is a recognized complication of left-sided infective endocarditis, with symptomatic events occurring in approximately 5–6% of patients in large prospective cohorts [72], while systematic abdominal CT imaging detects splenic lesions (including asymptomatic infarcts) in 19–34% of left-sided IE patients [77], and its presentation is highly variable. In cases of infectious presentation persisting despite appropriate treatment in splenic infarction, a diagnosis of splenic abscess must be highly suspected [78]. Septic pulmonary emboli and pulmonary abscesses are more commonly associated with right-sided IE and are typically seen in people who use intravenous drugs [79].

Heart failure, due to valvular damage, is the most common complication in IE patients and is seen in 25–30% of patients [34,72]. Although heart failure in those patients can vary in severity, nearly 38% present with New York Heart Association (NYHA) class IV symptoms, suggesting severe limitations in daily living [80]. The presence of heart failure in IE patients is also associated with higher mortality rates compared to IE patients without heart failure (29.7% and 13.1%, respectively) [80]. Mitral regurgitation may occur in IE patients due to mechanisms such as chordae tendineae rupture, leaflet perforation, or secondary to abscess formation [81]. Valve leaflet perforations or rupture, occurring in about 13–15% of IE patients based on large multicenter cohort data, may lead to severe valvular regurgitation and prompt early diagnosis and management [42,82,83]. IE may extend to the valves, causing perivalvular or periannular abscesses, a relatively common complication associated with high morbidity and mortality rates [84]. These abscesses are more common in PVEs than NVEs and may lead to conduction abnormalities, fistula formation, and heart failure [85,86]. Aortic valve endocarditis poses a higher risk for perivalvular abscess formation due to the anatomic proximity of the aortic annulus to the conduction system. The development of new conduction abnormalities, including atrioventricular block, should raise concerns for perivalvular extension and prompt urgent further imaging [72,87]. A rare but life-threatening complication in IE patients is mycotic aneurysms (MAs), caused by septic arterial embolization to the vasorum [33,88]. MAs carry a high risk of rupture due to the friability of the infected arterial walls, which may lead to the most feared complications of MAs, such as subarachnoid hemorrhage (SAH) or intracerebral hemorrhage (ICH) [89,90]. Rupture of MAs boasts a high mortality rate of 60–90% despite aggressive treatment, underscoring the value of prompt diagnosis and management of unruptured MAs [91,92].

## 6. Procedural Considerations in Infective Endocarditis

A significant risk of introducing bacteremia arises during dental procedures, particularly those involving gingival manipulation or perforation of the oral mucosa [93]. This transient bacteremia poses a great threat to patients with underlying cardiac conditions or prosthetic heart valves, as it can lead to the development of IE [93]. A 2025 meta-analysis reported a statistically significant association between IE and invasive dental procedures (OR 1.49, 95% CI 1.25–1.76; *p* < 0.00001) [94]. The AHA recommends consideration of IE antibiotic prophylaxis for high-risk patients undergoing dental procedures that only involve gingival manipulation [95]. Importantly, daily oral hygiene and maintenance of dental health likely play a greater role in reducing IE risk than antibiotic prophylaxis alone, as transient bacteremia more commonly arises from routine activities such as tooth brushing [2,95]. Table 1 summarizes the profile of high-risk patients who require antibiotic prophylaxis and the antibiotic choice.

The high-risk group includes patients with prosthetic cardiac valves, a history of IE, cardiac transplant with valve regurgitation due to an abnormal valve, unrepaired cyanotic congenital heart defect (CHD), or repaired CHD with residual shunts or valvular regurgitation [95]. It is recommended that dental procedures be delayed for at least 10 days after completion of a long-term antibiotic regimen, including those recommended for IE prophylaxis [96].

While dental procedures are most emphasized in the context of IE prophylaxis, recent large case-crossover and population-based studies have demonstrated a temporal association between IE and a range of nondental invasive procedures. These include cardiovascular procedures (such as device implantation and coronary interventions), gastrointestinal endoscopy, bronchoscopy, and procedures involving vascular access [97,98,99]. These associations are most pronounced in the weeks preceding IE diagnosis and are thought to reflect procedure-related bacteremia in susceptible individuals. However, the available evidence remains observational, and a causal relationship has not been definitively established.

Despite increased risk, routine antibiotic prophylaxis is not recommended for patients with bicuspid aortic valve or mitral valve prolapse in the absence of other high-risk features [66,95]. However, this classification remains debated; a large multicenter study demonstrated that IE in BAV and MVP patients is characterized by significantly higher rates of viridans group streptococcal infection and odontologic origin, with intracardiac complication rates comparable to those of high-risk patients, prompting calls to reconsider prophylaxis in these populations [100]. More recently, an increase in oral streptococcal IE among moderate-risk patients following the restriction of prophylaxis has been observed, particularly in those with congenital valve anomalies [101]. Taken together, these findings highlight an ongoing tension between guideline-based restriction of prophylaxis and emerging evidence suggesting a potentially higher risk profile in selected moderate-risk populations, underscoring the need for further prospective data to better inform prophylaxis strategies.

## 7. Management of Endocarditis

### 7.1. Antimicrobial Therapy

The cornerstone of acute IE management is the prompt initiation of high-dose intravenous empiric antibiotics, guided by the clinical setting (native vs. prosthetic valve, healthcare exposure, and IV drug use), Gram stain results if available, and local microbiologic resistance patterns [36]. Although definitive antimicrobial therapy in IE is organism-directed, initial empiric treatment is guided by clinical context, including valve type (native vs. prosthetic), healthcare exposure, and patient-specific risk factors such as intravenous drug use. This distinction is critical, as early therapy must provide adequate coverage before microbiologic data are available.

For example, in suspected native valve endocarditis, empiric regimens typically include vancomycin in combination with a third- or fourth-generation cephalosporin (e.g., ceftriaxone or cefepime) to provide coverage against *Staphylococcus aureus*, viridans group streptococci, and enterococci [102]. In contrast, prosthetic valve endocarditis or healthcare-associated infections require broader coverage due to increased likelihood of resistant organisms, often incorporating vancomycin plus cefepime with consideration of additional agents depending on timing of prosthesis implantation and local resistance patterns [66,102]. In people who inject drugs, empiric therapy should ensure adequate coverage for *Staphylococcus aureus* (including MRSA); however, because injection practices may involve nonsterile water, coverage for *Pseudomonas* spp. with cefepime should also be considered when Gram-negative organisms are suspected [52]. These empiric strategies should be promptly refined once microbiologic identification and susceptibility data become available. Table 2 summarizes organism-directed antimicrobial regimens for common causes of infective endocarditis; however, these should be interpreted within the clinical context, as empiric treatment strategies differ based on patient presentation and risk profile.

Empiric antibiotic therapy for acute native valve IE should begin promptly after obtaining blood cultures, guided by the most likely pathogens and patient-specific risk factors. According to the 2015 AHA/IDSA scientific statement, vancomycin in combination with a third-generation cephalosporin (e.g., ceftriaxone) is the preferred empiric regimen for most patients with native valve IE, especially those with risk factors for MRSA or enterococci [36]. This combination provides broad-spectrum coverage against the principal pathogens, *Staphylococcus aureus*, *Streptococcus* spp., and *Enterococcus* spp., and is widely applicable to both community-acquired and healthcare-associated infections. PVE represents a distinct clinical entity characterized by a higher burden of resistant organisms, biofilm-associated infection, and increased morbidity, often necessitating broader empiric antimicrobial coverage compared to native valve disease.

In contrast, the 2023 ESC guidelines recommend a more streamlined empiric approach with a vancomycin-based regimen but without routine gentamicin use, even in prosthetic valve IE, to minimize nephrotoxicity [66]. Gentamicin is reserved only for cases with documented synergy requirements, such as specific *Enterococcus faecalis* infections. Additionally, the ESC guidelines allow for oral step-down therapy and even ambulatory parenteral antibiotic therapy (OPAT) in select patients with stable disease and confirmed pathogen susceptibility [66], a concept not explicitly addressed in the AHA/IDSA guidance. This strategy is supported by the POET trial, which demonstrated that switching to oral antibiotic therapy after initial stabilization was noninferior to continued intravenous therapy with respect to a composite outcome of mortality, unplanned surgery, embolic events, or bacteremia relapse [103]. In POET, oral regimens included agents such as linezolid, amoxicillin–clavulanate, or a fluoroquinolone–rifampin combination, depending on pathogen susceptibility [103]. These data form part of the evidence base for the ESC recommendation for selected stable patients. However, the POET trial enrolled a highly selected population of clinically stable patients who had already responded to intravenous therapy, with a predominance of streptococcal infections and limited representation of higher-risk groups, which may limit the generalizability of its findings [103].

Once the causative organism and susceptibilities are known, definitive therapy should be narrowed. For MSSA, both guidelines recommend β-lactams (e.g., nafcillin or cefazolin) over vancomycin given their better bactericidal activity and clinical outcomes [104]. A randomized controlled trial also demonstrated that cefazolin may be non-inferior or even preferable to nafcillin due to better safety and lower nephrotoxicity, especially in older adults [105]. For *Enterococcus faecalis*, combination therapy with ampicillin plus ceftriaxone has been shown to be as effective as ampicillin–gentamicin but with a significantly better renal safety profile [106], and it is now preferred in both North American and European guidelines.

The duration of antibiotic therapy in IE is influenced by the causative organism, the presence of prosthetic material, and whether complications (e.g., abscess, emboli, heart failure) are present. According to both the 2015 AHA/IDSA statement and the 2023 ESC guidelines, native valve IE due to susceptible streptococci typically requires 4 weeks of intravenous antibiotics, while staphylococcal and enterococcal IE usually requires 6 weeks [36,66].

In PVE, longer treatment durations are standard: At least 6 weeks of antibiotics are generally required, and in some cases, therapy may be extended depending on complications or delayed surgical intervention [66]. For example, PVE due to *Staphylococcus aureus* often warrants 6 weeks of a combination regimen (e.g., vancomycin + rifampin ± gentamicin). Rifampin is initiated only after blood cultures have cleared to avoid resistance [36].

In culture-negative IE, management is guided by epidemiologic clues and targeted molecular or serologic diagnostics. Common culprits include *Coxiella burnetii*, *Bartonella* spp., and *Tropheryma whipplei*. Empiric regimens typically extend for ≥6 weeks and should be reassessed when additional diagnostic information becomes available [36,66,107].

Fungal endocarditis, though rare (1–10% of cases), carries a particularly high mortality rate and often affects immunocompromised hosts, prosthetic valve recipients, or patients with prolonged central venous access [108,109]. *Candida* spp. and *Aspergillus* spp. are the most common etiologies. Initial therapy typically includes liposomal amphotericin B or an echinocandin (e.g., micafungin), followed by step-down oral therapy such as fluconazole in susceptible isolates. Lifelong antifungal suppression may be necessary in certain cases. Surgical intervention is frequently required, given poor responses to medical therapy alone [36,108].

Close clinical monitoring, including follow-up transthoracic or transesophageal echocardiography, is recommended after completing antibiotic therapy, especially in patients with prosthetic valves, Enterococcus infections, or those who did not undergo surgery [5].

### 7.2. Surgical Therapy

Surgical intervention remains a cornerstone in the management of IE, with an estimated 25% to 50% of patients requiring valve surgery during their initial hospitalization [72,110,111,112]. The main indications for surgery include heart failure resulting from severe valvular dysfunction, uncontrolled infection such as perivalvular abscess or persistent bacteremia despite appropriate antibiotics, prevention of embolic events in the presence of large vegetations, and structural complications like prosthetic valve dehiscence or dysfunction [36,113]. Among these, heart failure is the most frequent indication and is associated with the greatest potential for mortality benefit when surgery is performed in a timely fashion.

Timing is critical, particularly in high-risk patients. Studies have demonstrated improved outcomes with early surgery, especially when performed within 7 days of diagnosis in patients with large vegetations (>10–15 mm) and prior embolic events or early signs of heart failure [114]. For example, the EASE trial showed that early surgery significantly reduced the composite risk of embolism or death at 6 months compared to conventional medical therapy [115]. Conversely, patients with intracranial hemorrhage or extensive ischemic stroke often require delayed surgery to mitigate perioperative neurologic risk, and the optimal timing in such cases remains a matter of clinical judgment [116].

The 2023 ESC guidelines emphasize early surgical consultation for all patients with poor prognostic markers or complications, recommending that a multidisciplinary endocarditis team, including infectious diseases specialists, cardiologists, neurologists, and cardiothoracic surgeons, be involved from the outset [66]. This team-based approach has been associated with reduced in-hospital mortality, fewer delays in surgery, and improved adherence to best practices [117]. The AHA/IDSA 2015 scientific statement also outlines surgical indications clearly but places less emphasis on the operational structure of multidisciplinary teams [36].

Surgical decision-making is especially complex in PVE, where reoperation poses technical and clinical challenges. Nonetheless, surgery is frequently indicated in PVE when imaging or intraoperative inspection reveals abscess formation, prosthesis loosening, or relapse of infection despite appropriate therapy [36,66]. These patients often have poorer outcomes than those with native valve IE, underscoring the importance of individualized surgical planning.

In contrast, right-sided IE presents distinct clinical features and management considerations. Right-sided IE, most commonly involving the tricuspid valve, represents a distinct clinical entity, frequently associated with IVDU and characterized by a higher prevalence of *Staphylococcus aureus* infection [2,118]. Management is often primarily medical; however, intervention may be required in cases of persistent bacteremia, large vegetations (≥20 mm), particularly in the setting of recurrent pulmonary emboli, or severe tricuspid regurgitation with right heart failure [36,52]. In selected patients who are poor surgical candidates, percutaneous approaches such as the AngioVac system have emerged as potential alternatives for vegetation debulking, although data remain limited and largely derived from observational studies [119,120]. These approaches may reduce bacterial burden and facilitate clinical stabilization, but they do not treat underlying valve dysfunction and may unmask or exacerbate tricuspid regurgitation; moreover, residual vegetation is commonly present post-procedurally, and the technique does not address valvular abscesses or leaflet destruction [52,119]. Accordingly, percutaneous mechanical aspiration is best conceptualized as a bridging strategy to clinical stabilization or definitive surgical intervention, and its role relative to conventional surgery continues to evolve [119,121].

## 8. Knowledge Gaps and Future Directions

Despite advances in the diagnosis and management of IE, several important knowledge gaps remain. The evidence supporting many clinical decisions, particularly surgical indications, is largely derived from observational studies, as the number of well-conducted randomized controlled trials in IE remains limited [2,36]. Consequently, the relative strength of evidence varies across specific indications, and the optimal timing of surgery, especially in patients with neurologic complications or large vegetations without prior embolic events, remains an area of ongoing debate [2,115,122,123]. Recent guideline updates further reflect this evolving evidence base, suggesting that surgery should not be delayed following ischemic stroke when otherwise indicated, while hemorrhagic stroke may warrant a delay depending on clinical stability [36,66].

Similarly, many recognized risk factors for IE represent epidemiologic associations rather than clearly established causal relationships, as demonstrated in large prospective cohort studies [5]. As the epidemiology of IE continues to evolve, particularly with the increasing use of prosthetic valves, transcatheter valve interventions, and CIEDs, further work is needed to refine risk stratification and better define high-risk populations [1,44]. The formal incorporation of prosthetic material, transcatheter interventions, and device-related infections into contemporary diagnostic frameworks underscores the shifting clinical landscape and rising burden of healthcare-associated IE.

Reported complication rates, including embolic events, heart failure, and conduction abnormalities, vary widely across studies, reflecting differences in patient populations, diagnostic approaches, and study design [5,74]. This variability underscores the need for standardized definitions and contemporary prospective data to more accurately characterize disease burden and outcomes.

Antimicrobial management strategies also demonstrate variability across international guidelines, reflecting differences in local resistance patterns, healthcare infrastructure, and interpretation of available evidence [33,36,66]. Although studies such as the POET trial support oral step-down therapy in selected patients, the generalizability of these approaches to broader and more complex populations remains uncertain. In particular, the POET trial enrolled a highly selected cohort with a predominance of streptococcal infections and limited representation of higher-risk groups, including *Staphylococcus aureus* infection, prosthetic valve endocarditis, and patients with complications requiring surgery [103]. Real-world implementation studies further highlight that only a subset of eligible patients can safely undergo oral step-down therapy, emphasizing the need for additional randomized data in more diverse populations.

Advances in microbiologic diagnostics, including molecular techniques and metagenomic next-generation sequencing, offer promise in improving pathogen detection, particularly in culture-negative IE [45,48]. However, their integration into routine clinical practice remains limited by cost, availability, and lack of standardized interpretation frameworks. Although emerging data demonstrate improved diagnostic yield compared with conventional culture-based methods, the clinical application of these technologies requires multidisciplinary expertise and further validation in prospective studies.

Future research should focus on generating high-quality randomized data to guide surgical decision-making, refining individualized antimicrobial strategies, improving diagnostic accuracy through integration of advanced imaging and molecular tools, establishing standardized frameworks for interpretation of molecular diagnostics, and developing validated risk prediction models to support personalized, multidisciplinary care.

## 9. Conclusions

IE continues to pose diagnostic and therapeutic challenges due to its heterogeneous presentations, evolving microbiologic profile, and the complexity of treatment decisions. The revised diagnostic criteria, most notably the Duke-ISCVID 2023 update, have refined case definitions to account for culture-negative cases, intraoperative findings, and modern imaging modalities, thereby enhancing diagnostic accuracy in difficult cases such as prosthetic valve and device-associated infections.

Advances in microbiologic techniques, including serologic testing, molecular diagnostics, and next-generation sequencing, have expanded our ability to detect fastidious or previously unrecognized pathogens. These innovations are particularly valuable in cases of culture-negative endocarditis, where traditional methods fall short.

Therapeutically, early and appropriately targeted antibiotic therapy remains the cornerstone of treatment. Guideline-directed regimens vary slightly between international societies, with recent European recommendations offering broader endorsement of oral step-down therapy and outpatient antibiotic management in select patients. Duration of therapy depends on pathogen, valve type, and disease complications, with prosthetic valve infections typically requiring extended treatment. In culture-negative and atypical infections, therapy is individualized, prolonged, and increasingly guided by molecular diagnostics.

Surgical intervention is required in up to half of IE cases, most commonly for heart failure, uncontrolled infection, or to prevent embolic events. Evidence supports early surgery in high-risk patients with large vegetations or hemodynamic compromise, while neurologic complications may necessitate delayed intervention. The adoption of multidisciplinary endocarditis teams has demonstrated improved outcomes, underscoring the need for coordinated, individualized care.

Ultimately, optimal outcomes in IE hinge on a high index of suspicion, early diagnosis, integration of modern microbiologic tools, timely antimicrobial and surgical intervention, and longitudinal follow-up, particularly in high-risk populations such as those with prosthetic valves, culture-negative disease, or fungal endocarditis. As the epidemiology of IE continues to shift, clinical pathways must adapt accordingly to ensure comprehensive, guideline-driven, and patient-centered management.

## Figures and Tables

**Table 1:** Endocarditis prophylaxis for high-risk patients and dental procedures.

Indications for antibiotic prophylaxis before at-risk dental procedures ^a^
Prosthetic cardiac valve, including transcatheter valve, or prosthetic material used for cardiac valve repair
Previous IE
Unrepaired cyanotic CHD
Within six months following complete repair of CHD with prosthetic material or device
Repaired CHD with residual defects at the site or adjacent to the site of a prosthetic device/patch
Cardiac transplant recipients who develop cardiac valvopathy

Recommended prophylaxis: Amoxicillin 2 g orally 30–60 min before procedure. Penicillin allergy: azithromycin or clarithromycin 500 mg orally 30–60 min before procedure. ^a^ No prophylaxis is recommended for routine anesthetic injections, radiographs, orthodontic placement, or shedding of primary teeth.

**Table 2:** Recommended treatment regimens for common causes of endocarditis *.

Pathogen	Valve Type	Preferred Regimen	Duration	Notes
Viridans group Streptococci	Native (Pen-S)	Penicillin G (12–18 million U/day IV q4–6h) or ceftriaxone 2g IV q24h ± gentamicin	4 weeks (no gentamicin) 2 weeks (with gentamicin)	Gentamicin only if CrCl > 20, uncomplicated cases
	Prosthetic	Penicillin G ± gentamicin or ceftriaxone ± gentamicin	6 weeks	Gentamicin optional in susceptible strains; consider based on MIC and clinical scenario
*Streptococcus gallolyticus*	Native/prosthetic	Same as viridans streptococci	4–6 weeks	Colonoscopy recommended given association with colon
*Staphylococcus aureus* (MSSA)	Native	Nafcillin/oxacillin or cefazolin	6 weeks	Cefazolin preferred if penicillin allergy or renal insufficiency
	Prosthetic	Nafcillin/oxacillin + rifampin + gentamicin	≥6 weeks	Rifampin typically initiated after blood culture clearance
*Staphylococcus aureus* (MRSA)	Native	Vancomycin	≥6 weeks	Daptomycin if vancomycin-intolerant
	Prosthetic	Vancomycin + rifampin ± gentamicin	≥6 weeks	
*Enterococcus faecalis* (susceptible)	Native/prosthetic	Ampicillin + ceftriaxone	6 weeks	Preferred regimen due to renal safety
		Ampicillin + gentamicin	4–6 weeks	Only if CrCl >50 and gentamicin-susceptible
Enterococcus (HLAR)	Native/prosthetic	Ampicillin + ceftriaxone	6 weeks	
HACEK organisms	Native/prosthetic	Ceftriaxone or ampicillin or ciprofloxacin	4 weeks (native) 6 weeks (prosthetic)	
Culture-negative IE	Native	Vancomycin + cefepime (acute) or vancomycin + ampicillin-sulbactam (subacute)	≥4–6 weeks (depending on organism and clinical course)	Regimen guided by clinical presentation: acute = coverage for *Staphylococcus aureus* and aerobic Gram-negative organisms. Subacute = coverage for viridans group streptococci (VGS), HACEK organisms, and enterococci. Adjust based on molecular/serologic testing
	Prosthetic	Vancomycin + cefepime or piperacillin–tazobactam ± rifampin ± gentamicin (early, ≤1 year) or Vancomycin + ceftriaxone (late, >1 year)	≥6 weeks	Early PVE: consider addition of rifampin ± gentamicin for staphylococcal and nosocomial pathogen coverage. Late PVE: pathogen profile similar to native valve endocarditis. Adjust based on diagnostic results
Fungal IE (e.g., Candida)	Native/prosthetic	Liposomal amphotericin B ± flucytosine or high-dose echinocandin	≥6 weeks + prolonged or suppressive fluconazole therapy in selected patients	Lifelong suppression may be required in prosthetic valve IE or non-surgical candidates; surgical intervention is typically recommended

HLAR = High-level aminoglycoside resistance. * These regimens are intended as illustrative summaries and do not substitute for guideline-directed recommendations or consideration of local resistance patterns, patient-specific factors, and clinical context. Specific regimen selection, duration, and adjunctive therapies (e.g., gentamicin or rifampin) may vary depending on organism susceptibility, valve type, and clinical scenario.

## Data Availability

No new data were created or analyzed in this study.
